# Clinical features of bipolar disorder in adolescents with intellectual disability

**DOI:** 10.1192/j.eurpsy.2021.1692

**Published:** 2021-08-13

**Authors:** M. Hafnaoui, R. Gadhoum, Z. Abbes, M. Hajri, S. Jelili, S. Halayem, A. Bouden

**Affiliations:** Child And Adolescent Psychiatry Department, RAZI HOSPITAL, Mannouba, Tunisia

## Abstract

**Introduction:**

Bipolar disorder in children and adolescents is distinguished by a variable and complex clinical expression. Mood is difficult to assess, mood symptoms are often masked and signs of disorganization may be in the limelight. This can be more difficult when adolescents have intellectual disability (ID).

**Objectives:**

This work aims to describe diagnostical and therapeutical features of bipolar disorder in adolescents with ID.

**Methods:**

Case reports about five patients who have been diagnosed with bipolar disorder associated to ID, all seen and treated in child and adolescent psychiatry department of Razi Hospital, in Tunis.

**Results:**

The study focused on three girls and two boys, all with mild to moderate ID. Four patients had psychiatric family history of bipolar disorder and ID. Only one patient was followed since childhood for mixed ADHD. The average age of onset of bipolar disorder was 14 years. Four cases were inaugurated by manic access; the fifth was a depressive disorder followed by a manic shift under sertraline. Only one case was rapidly favorable, under 10mg of Olanzapine, without any recurrence or relapse during 18 months of follow-up. Another case was slower but also favorable, under 10mg of Olanzapine. We found resistance to usual treatments for 2 patients; these did not evolve well under conventional thymoregulators, or different antipsychotic molecules, nor with combinations of two thymoregulators + an antipsychotic. One of them benefited from a combination of clozapine and lithium with excellent response.

**Conclusions:**

Bipolar disorder comorbid with ID in adolescents is a difficult diagnostic entity and particularly hard to manage.

**Disclosure:**

No significant relationships.

